# Successful Weaning off LVAD Support in an Infant by Pulmonary Artery Banding

**DOI:** 10.1055/a-2564-2280

**Published:** 2025-06-10

**Authors:** Maria Jaros, Jelena Pabst von Ohain, Marcus Fischer, Nikolaus Alexander Haas

**Affiliations:** 1 Kinderkardiologie und Pädiatrische Intensivmedizin, LMU Klinikum Abteilung für Kinderkardiologie und Pädiatrische Intensivmedizin, Munich, Germany; 2 Sektion für Chirurgie angeborener Herzfehler und Kinderherzchirurgie der Herzchirurgischen Klinik, Ludwig Maximilian University of Munich, Munchen, Bayern, Germany; 3 Ludwig Maximilians University Munich, Munchen, Germany; 4 Department of Pediatric Cardiology and Pediatric Intensive Care, Ludwig-Maximilians-Universitat Munchen Medizinische Fakultat, Munchen, Germany

**Keywords:** pulmonary artery banding, berlin heart, LVAD, left ventricular assist device, weaning, myocarditis in children

## Abstract

**Background:**

Cardiomyopathy is the leading indication for transplantation in children. Mechanical cardiac support systems play a significant role in treating severe pediatric heart failure for cardiomyoptahy or myocarditis either for recovery or as bridge to transplant. In most cases of chronic heart failure extending over the acute inflammatory phase transplantation is necessary. Organ shortage results in a necessity for further treatment options in terminal heart failure. A new and controversially discussed approach to treat failing (left) ventricles includes pulmonary banding; In some observational studies pulmonary artery banding was performed in patients with severe (left) heart failure and adequate function of the right ventricle. The effect is postulated by improving the contra-lateral (left) ventricular function with intraventricular cross-talk and subsequent myocardial changes. Whereas selected patients may however benefit from PA banding with subsequent training of the left ventricle, nevertheless this strategy is controversially discussed.

**Case Description:**

A 3-year-old girl with inflammatory myocarditis required left ventricular assist device (LVAD, Berlin Heart)) support. After repetitive weaning failure, pulmonary artery (PA) banding was performed during LVAD support that resulted in an improvement of the left ventricular function and finally LVAD explantation.

**Conclusion:**

Selected patients may benefit from PA banding with subsequent training of the left ventricle even in the setting of LVAD.

This case here is the first reported case where PA banding was successful in the weaning process of a child on mechanical circulatory support (MCS). This principle of pulmonary banding to improve left ventricular function in severe heart failure can apparently also be applied to patients on left ventricular assist devices (LVAD).

## Introduction

Cardiomyopathy is the leading indication for transplantation in children. Ten percent of cases of dilated cardiomyopathy are caused by acute myocarditis. 1 Myocarditis is an inflammation of cardiac myocytes, causing myocardial injury. 2 The leading cause in children is infectious etiology, today most commonly parvovirus B 19. 3 Acute myocarditis can lead to borderline systolic function and arrhythmias. The progress into inflammatory cardiomyopathy with massive dilation of the left ventricle is possible where spontaneous recovery is not as likely anymore. 4

Phosphodiesterase inhibitors like milrinone, levosimendan (Ca-sensitizers), or even catecholamines are commonly used as first-line therapy for ionotropy. 5 In addition oral heart failure therapy should be initiated once the patient is beyond the acute stage but still continues to have a compromised systolic function. 2

According to Schranz et al., there is a newer and controversially discussed approach to the treatment of severe (left-sided) heart failure, some forms of cardiomyopathy, and myocarditis which includes the modification of the right ventricular afterload by pulmonary banding, inducing right ventricular hypertrophy and thereby improving the contra-lateral ventricular function by so far unknown interventricular cross-talk mechanisms. 6 7

To assess ventricular failure, today brain natriuretic peptide (BNP) values are determined regularly. Due to the age dynamics; however, the absolute values have not been reliable. 8 Palm et al reinvented the BNP value by introducing the zlog value of NT-proBNP. zlog-proBNP (the zlog-is a value as basis for the standardization of laboratory results. It describes the standardized range [−1.96 to +1.96] of the normal reference values according to age, size, weight etc.) levels can be interpreted as low (≤1.0), normal (>1.0 and ≤1.96), intermediate (>1.96 and ≤ 3.0), and high (>3.0). 9 10 Cardiac recovery may be assessed by normalization of BNP levels even during assist device therapy in adults. 9

We present a case in which pulmonary artery (PA) banding was used to enable and support left ventricular recovery after failed recovery by LVAD (left ventricular assist device) implantation alone. The timely course of recovery could be monitored by BNP zlog values in addition to echocardiography.

## Case

The patient was born at term with no prior known diseases. There is no positive family history. She presented at our department with progressive cardiomyopathy, diagnosed in her home country (Romania) and already treated with oral anti-congestive heart failure therapy. (i.e., ACE inhibitors [angiotensin converting enzyme], β blockers, and antidiuretics).

**Fig. 1:**
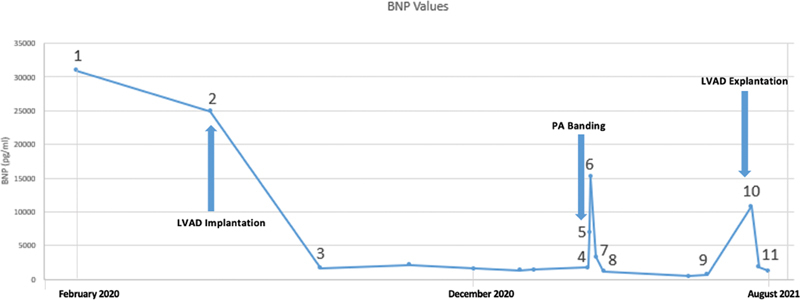
Absolute BNP values; arrow shows the time of PA banding; the graph shows the timeline from the first presentation until the week after the LVAD explantation. It shows elevated BNP values in the beginning and during the time of PA banding with a drastic drop after surgery. (1) First BNP after presentation; (2) 6 days after LVAD implementation; (3) 3 months after LVAD implementation; (4) PA Banding; (5) 1 day after debanding; (6) 2 days after debanding; (7) 4 days after partial debanding; (8) 10 days after partial debanding; (9) 3 months after partial debanding; (10) 1 day after LVAD explantation; and (11) 1 week after LVAD explantation.

**Fig. 2:**
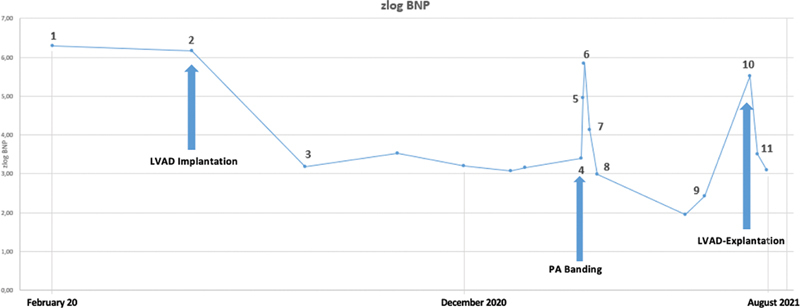
zlog values; the graph shows the timeline from the first presentation until the week after the LVAD explantation. It shows elevated zlog BNP values in the beginning and during the time of PA banding with a drastic drop after surgery. (1) First BNP after presentation; (2) 6 days after LVAD implementation; (3) 3 months after LVAD implementation; (4) PA Banding; (5) 1 day after debanding; (6) 2 days after debanding; (7) 4 days after partial debanding; (8) 10 days after partial debanding; (9) 3 months after partial debanding; (10) 1 day after LVAD explantation; and (11) 1 week after LVAD explantation.

The first cardiac catheter was performed 2 months after the first presentation (at the age of 1 year) and the biopsy revealed a parvovirus B19-associated myocarditis. The progression of the disease led to severe cardiac decompensation within the next 6 months. The BNP increased accordingly. ( Figs. 1 and 2 )

Therapy was first increased by milrinone and levosimendan, but when the systolic function continued to worsen and multi-organ impairment developed, an LVAD—extracorporal assist device was implanted at the age of 1.5 years. (i.e., Berlin heart excor, LVAD 25 ml ventricle). Subsequently and after initial stabilization, several attempts to wean the young patient off the Berlin Heart failed due to left ventricular enlargement and clinical deterioration. In the meantime, Interferon therapy was implemented for 6 months while on the LVAD, however with no significant benefit.

As the right ventricle continuously showed excellent function, pulmonary banding was deemed a potential beneficial therapy. Therefore, before deciding to list her for transplantation and after extensive discussion with the parents, a pulmonary banding was performed 10 months after the LVAD implementation.

The initial banding was placed with a 4 mm residual lumen (∼30% diameter) in the middle of the pulmonary artery. It was fixated when the right ventricle had a systolic pressure of 60 mmHg and the left ventricle 85 mmHg. The echocardiography showed good cardiac function and a systolic gradient of 46 mm Hg over the PA banding.

**Fig. 3:**
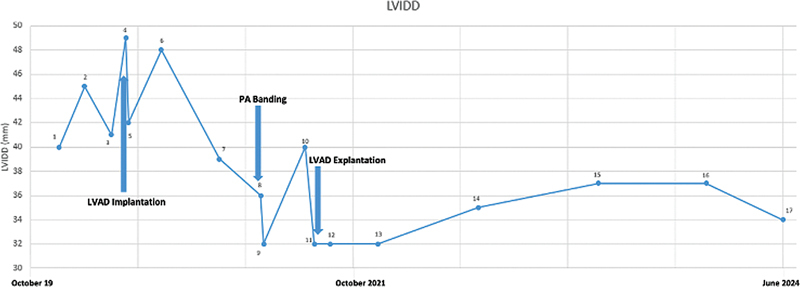
LVIDD in mm; arrow shows the time of PA banding: (1) first presentation; (2) first heart catheter; (3) start decompensation; (4) 4 days before LVAD implantation; (5) 1 day after LVAD implantation, (6) 2.5 months after LVAD implantation, (7) 7 months after LVAD implantation, (8) 3 days after partial debanding, (9) 2 weeks after partial debanding, (10) 3 months after partial debanding, (11) 4 days LVAD explantation, (12) 1 month after LVAD explantation, (13) 6 months after LVAD explantation, (14) 1 year after LVAD explantation, (15) 2 years after LVAD explantation, (16) 3 years after LVAD explantation, and (17) 3.5 years after LVAD explantation.

Within 48 hours the patient however developed a ventricular tachycardia and had to be resuscitated. The right heart then showed significant dilatation on echocardiography in the performed echocardiography with poor contraction and the gradient over the pulmonary artery banding (PAB) was 20 mmHg only. Apparently, the initial banding was too tight and the indication for partial debanding to relieve the right ventricle was given. The PA banding was released operatively to an 8 mm lumen (∼50% diameter) and the RV (right ventricle) function recovered rapidly. Echocardiographic follow-up showed a maximal gradient of approximately 25 mm Hg. Subsequently, the cardiac function improved further and 6 days after surgery the catecholamine support could be terminated. Over the next weeks, the left ventricular function improved constantly, the LVIDD (left ventricular internal dimension in diastole) slowly recovered and 3 months later the LVAD could be weaned. ( Fig. 3 )

In addition, the BNP decreased after the partial debanding ( Figs. 1 and 2 ) After LVAD explanation, the heart failure treatment could be reduced to β blockers and ace inhibitors while the diuretic treatment was terminated. During the follow-up care now for the past 4 years, the function of the left ventricle is normalized, the patient showed no clinical heart failure signs and everyday life can be managed well.

## Discussion

It is well known that children with myocarditis have a better outcome than those with an (early) onset of dilative cardiomyopathy. 4 Twenty-three percent of patients with severe myocarditis in the United States need mechanical circulatory support (MCS) via extracorporeal membrane oxygenation (ECMO) or ventricular assist device (VAD) in the early phase of the disease. If chronic heart failure precedes the acute inflammatory phase, transplantation is often necessary. 11

The waiting times for an organ in countries such as the United States are significantly shorter (median time of 32 days) as compared with Germany where a mean waiting time is more than 1 year. 4 12 In Europe in general and especially in Germany there is a shortage of organs due to regulatory effects which makes the MCS support more relevant for many children with acute and severe heart failure. 13 14 Longer waiting periods on MCS inadvertently offer the possibility that some of these patients can be weaned off the MCS with satisfying results. 15

Due to longer waiting times on the transplant list, it may be relevant and necessary to evaluate other treatment options, which is the reason why PAB comes into play. As described in the work by Schranz et al., heart transplantation (Htx) seems like the only lifesaving option for children with dilated cardiomyopathy. 7 New treatment options can be achieved by paradigm-shifting traditional heart failure therapies like modifying right ventricular afterload which then leads to a secondary RV hypertrophy and a postulated ventricular crosstalk and subsequent improvement in the left ventricular function. This can be achieved by PAB in young children.

In an initial case report by Schranz et al., an approximately 2-month-old patient with idiopathic dilated cardiomyopathy showed significant improvements after a PAB was performed. The left ventricular diameter decreased from 40 to 31 mm and the BNP levels decreased to normal values 6 weeks after the performance. 16

In a subsequent retrospective single-center observational study by Schranz et al., 12 children were observed after PAB was placed in patients with severe cardiomyopathy which showed improvement of the left ventricular function by ventricular crosstalk. 17

As indicated earlier, one of the theories behind this treatment strategy is due to a postulated inter-ventricular crosstalk the left ventricle can be improved, which makes the function of the right ventricle crucial in this method. 7

Ventricular arrhythmias are one of the possible complications that can appear after PA banding. 18

In general, MCS support reduces the mortality on the waiting list for transplants. However, according to a report by Euromacs, only 51% of children were transplanted after being 24 months on MCS support. Due to this limitation further treatment options remain important. 19 In the analysis of pedimacs, 55% of children developed acute right-sided heart failure after LVAD implantation. 20 Therefore, an excellent function of the RV is crucial when considering PA banding for LV (left ventricle) failure. Acute right ventricular failure is one of the main complications of LVAD implantation which would disqualify PAB as a treatment option. Regular follow-ups on the right ventricular function are therefore necessary and usually RV function improves over time. 21

There are animal studies that support this approach; 4-month-old sheep with doxorubicin-induced cardiomyopathy showed significant improvement in the LV functional diameters 3 months after PA banding. 22 In one of the first human case series the improvement was observed after 6 months and in neonates even after 2 to 6 weeks. 6 16

So far there is little evidence or even research regarding the possibility of PA banding for children on MCS support. Our case also shows the applicability also for this group of patients. However, a close monitoring of the right ventricle is necessary. This can be achieved by additional measurement of BNP levels. In our case, the zlog values stayed over 3 until banding was performed, which then caused a drop below 3 which correlates with a normal to intermediate risk. This classifies the explanation as a success.

## Conclusion

This case may illustrate that in selected patients PA banding with subsequent training of the right ventricle may also be applied in patients on left ventricular assist devices for severe LV heart failure enabling weaning off mechanical support over time.

## References

[1] Lipshultz S E Law Y M Asante-Korang A Cardiomyopathy in children: classification and diagnosis: a scientific statement from the American Heart Association Circulation 2019 140 01 e9 e68 31132865 10.1161/CIR.0000000000000682

[2] Williams J L Jacobs H M Lee S Pediatric myocarditis Cardiol Ther 2023 12 02 243 260 36906691 10.1007/s40119-023-00309-6 PMC10008072

[3] Esmel-Vilomara R Dolader P Izquierdo-Blasco J Parvovirus B19 myocarditis in children: a diagnostic and therapeutic approach Eur J Pediatr 2022 181 05 2045 2053 35138467 10.1007/s00431-022-04406-x

[4] Foerster S R Canter C E Cinar A Ventricular remodeling and survival are more favorable for myocarditis than for idiopathic dilated cardiomyopathy in childhood: an outcomes study from the Pediatric Cardiomyopathy Registry Circ Heart Fail 2010 3 06 689 697 20833772 10.1161/CIRCHEARTFAILURE.109.902833

[5] Deutsche Gesellschaft für Pädiatrische Kardiologie. Accessed April 11, 2025 at:https://www.dgpk.org/wp-content/uploads/ll_chronische_herzinsuffizienz_nov_2015_rickers.pdf

[6] Schranz D Krause U Kerst G Esmaeili A Paul T Functional regeneration of dilated cardiomyopathy by transcatheter bilateral pulmonary artery banding: first-in-human case series Eur Heart J Case Rep 2023 7 02 ytad052 36845833 10.1093/ehjcr/ytad052 PMC9954967

[7] Schranz D Recla S Malcic I Kerst G Mini N Akintuerk H Pulmonary artery banding in dilative cardiomyopathy of young children: review and protocol based on the current knowledge Transl Pediatr 2019 8 02 151 160 31161082 10.21037/tp.2019.04.09 PMC6514280

[8] Nir A Lindinger A Rauh M NT-pro-B-type natriuretic peptide in infants and children: reference values based on combined data from four studies Pediatr Cardiol 2009 30 01 3 8 18600369 10.1007/s00246-008-9258-4

[9] Palm J Holdenrieder S Hoffmann G Predicting major adverse cardiovascular events in children with age-adjusted NT-proBNP J Am Coll Cardiol 2021 78 19 1890 1900 34736565 10.1016/j.jacc.2021.08.056

[10] Palm J Hoffmann G Klawonn F Continuous, complete and comparable NT-proBNP reference ranges in healthy children Clin Chem Lab Med 2020 58 09 1509 1516(CCLM) 32305952 10.1515/cclm-2019-1185

[11] American Heart Association Pediatric Heart Failure and Transplantation Committee of the Council on Lifelong Congenital Heart Disease and Heart Health in the Young and Stroke Council Law Y M Lal A K Chen S Diagnosis and management of myocarditis in children: a scientific statement from the American Heart Association Circulation 2021 144 06 e123 e135 34229446 10.1161/CIR.0000000000001001

[12] Singh T P Almond C S Piercey G Gauvreau K Current outcomes in US children with cardiomyopathy listed for heart transplantation Circ Heart Fail 2012 5 05 594 601 22899768 10.1161/CIRCHEARTFAILURE.112.969980

[13] Barthmann M Organ Transplantation: German Reference Centre for Ethics in the Life SciencesAccessed May 19,2023at:https://www.drze.de/en/research-publications/in-focus/organ-transplantation

[14] Schranz D Akintuerk H Voelkel N F ‘End-stage’ heart failure therapy: potential lessons from congenital heart disease: from pulmonary artery banding and interatrial communication to parallel circulation Heart 2017 103 04 262 267 28011759 10.1136/heartjnl-2015-309110 PMC5293839

[15] Loebe M Hennig E Müller J Spiegelsberger S Weng Y Hetzer R Long-term mechanical circulatory support as a bridge to transplantation, for recovery from cardiomyopathy, and for permanent replacement Eur J Cardiothorac Surg 1997 11 S18 S24 9271176

[16] Schranz D Veldman A Bartram U Michel-Behnke I Bauer J Akintürk H Pulmonary artery banding for idiopathic dilative cardiomyopathy: a novel therapeutic strategy using an old surgical procedure J Thorac Cardiovasc Surg 2007 134 03 796 797 17723838 10.1016/j.jtcvs.2007.04.044

[17] Schranz D Rupp S Müller M Pulmonary artery banding in infants and young children with left ventricular dilated cardiomyopathy: a novel therapeutic strategy before heart transplantation J Heart Lung Transplant 2013 32 05 475 481 23410738 10.1016/j.healun.2013.01.988

[18] Panaioli E Khraiche D Pontailler M Prophylactic pulmonary artery banding in pediatric dilated cardiomyopathy: an additional therapeutic option J Cardiovasc Dev Dis 2024 11 03 79 38535102 10.3390/jcdd11030079 PMC10970918

[19] Donné M De Pauw M Vandekerckhove K Bové T Panzer J Ethical and practical dilemmas in cardiac transplantation in infants: a literature review Eur J Pediatr 2021 180 08 2359 2365 33959817 10.1007/s00431-021-04100-4

[20] Simpson K E Kirklin J K Cantor R S Right heart failure with left ventricular assist device implantation in children: an analysis of the Pedimacs registry database J Heart Lung Transplant 2020 39 03 231 240 31926747 10.1016/j.healun.2019.11.012

[21] Iacobelli R Di Molfetta A Brancaccio G Acute and long-term effects of LVAD support on right ventricular function in children with pediatric pulsatile ventricular assist devices ASAIO J 2018 64 01 91 97 28509675 10.1097/MAT.0000000000000596

[22] Yerebakan C Boltze J Elmontaser H Effects of pulmonary artery banding in doxorubicin-induced left ventricular cardiomyopathy J Thorac Cardiovasc Surg 2019 157 06 2416 2.428E7 30975548 10.1016/j.jtcvs.2019.01.138

